# Histological Changes Related to Symptomatic Improvement of Spontaneous Keloids Treated with a Low-Dosage Regimen of UVA-1 Phototherapy

**DOI:** 10.3390/dermatopathology7030009

**Published:** 2020-12-03

**Authors:** Carlos Cuenca-Barrales, José Aneiros-Fernández, Israel Pérez-López, Julia Rodríguez-Pérez, Ricardo Ruiz-Villaverde, Jorge Luis Espelt-Otero

**Affiliations:** 1 Dermatology Department, Hospital Universitario San Cecilio, 18016 Granada, Spain; ipl_elmadrono@hotmail.com (I.P.-L.); ismenios@hotmail.com (R.R.-V.); jespelto@hotmail.com (J.L.E.-O.); 2 Pathology Department, Hospital Universitario San Cecilio, 18016 Granada, Spain; janeirosf@hotmail.com; 3 Nursing Department, Hospital Universitario San Cecilio, 18016 Granada, Spain; jurope1958@hotmail.com

**Keywords:** pathology, clinical, keloid, phototherapy

## Abstract

Keloids are a difficult-to-treat disease characterized by an imbalance in mechanisms of tissue reparation. We present the case of a middle-aged woman with spontaneous keloids which histologically and clinically improved after UVA-1 phototherapy treatment. There are few reported cases of keloids treated with high doses of UVA-1 phototherapy. We used a low-dosage regimen with a good response in only one cycle, which could diminish the risk of skin cancer development.

## 1. Introduction

Keloids represent a form of abnormal wound healing, characterized by local fibroblast proliferation and excessive collagen production due to a loss of control in regulatory mechanisms of tissue reparation. Multiple treatments have been reported for this pathology, such as local corticosteroids, fluorouracil, imiquimod, silicone gel, cryotherapy, radiation and laser therapy; however, many of them are not effective enough and recurrences are frequent [1].

## 2. Case Report

**Figure 1:**
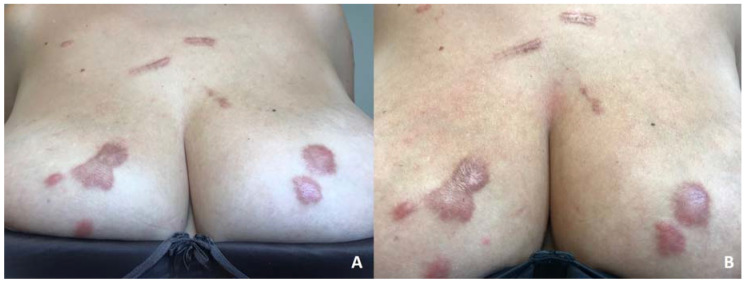
Spontaneous keloids on the chest and breasts of the patient before (**A**) and after (**B**) the treatment with UVA-1 phototherapy.

A healthy middle-aged woman with Fitzpatrick skin type III presented at our dermatologic outpatient clinic complaining of keloids on her back, chest and breasts. She had no family history of keloids and reported that these lesions had been spontaneously appearing since she was 15 years old; the latest one appeared two years ago on her breasts and were still growing (Figure 1A). 

**Figure 2:**
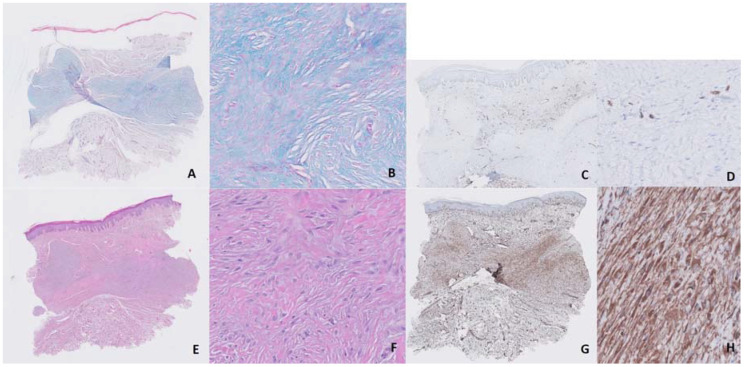
Before treatment, we observed basal deposits of mucin (**A**,**B**, 10× and 40×, respectively, alcian blue staining), few vascular structures (**C**,**D**, 10× and 40×, respectively, CD34 staining), disorganized collagen (**E**,**F**, 10× and 40×, respectively, hematoxylin–eosin staining) and a rich cellularity (**G**,**H**, 10× and 40×, respectively, vimentin staining).

**Figure 3:**
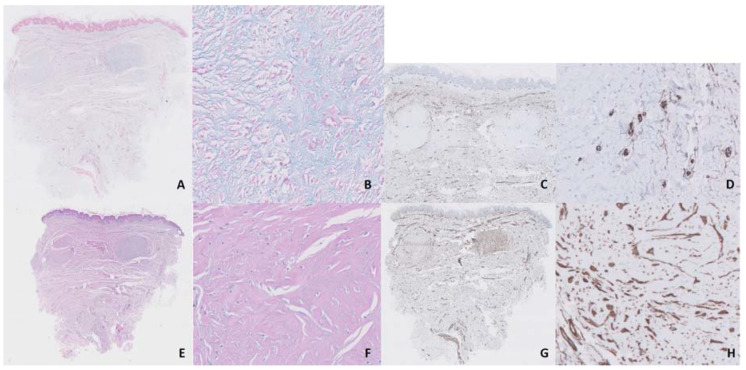
After treatment with a low-dosage regimen of UVA-1 phototherapy, we observed a reduction in mucin (**A**,**B**, 10× and 40×, respectively, alcian blue staining), neoangiogenesis (**C**,**D**, 10× and 40×, respectively, CD34 staining), denser and less disorganized collagen (**E**,**F**, 10× and 40×, respectively, hematoxylin–eosin staining) and a reduction in cellularity (**G**,**H**, 10× and 40×, respectively, vimentin staining) in comparison with Figure 2.

Several treatments with topical and intralesional corticosteroids and intralesional bleomycin had not been effective. We started treatment with UVA-1 phototherapy at 370 nm in a low-dosage regimen (starting dosage of 5 J/cm^2^, 3 sessions/week, with an increase of 5% in each session and a maximum dose of 15.95 J/cm^2^). After 25 sessions, with a total accumulated dose of 237.5 J, we observed an important reduction in pruritus and discomfort reported by the patient and a flattening and softening of keloids, although erythema response was limited and there was no significant aesthetic improvement (Figure 1B). Moreover, this response was maintained 3 months after treatment discontinuation. Histologic comparison between samples taken from the same keloid on the breast before and after treatment showed significant improvement, with denser and less disorganized collagen, a reduction in mucin and cellularity (after counting 10 mm^2^, we observed a reduction from 350 to 265 cells/mm^2^ after treatment with UVA-1 phototherapy) and neoangiogenesis (after counting 10 mm^2^, we observed an increase in vascular structures from 7 to 12/mm^2^ after treatment with UVA-1 phototherapy) (Figure 2 and Figure 3).

The case presented follows the Declaration of Helsinki. As this is a single, completely anonymous case, the approval of the Institutional Review Board was not required. The patient gave her informed consent.

## 3. Discussion

UVA-1 is a modality of phototherapy with a long wavelength, which allows it to achieve greater penetration and makes it especially effective for the treatment of sclerotic skin diseases [2]. This longer wavelength is also associated with a lower risk of burning during treatment than other phototherapy modalities. The main mechanism of action is the activation of fibroblasts, which produce metalloproteinases, with the consequent reduction in collagen production [3]. Other mechanisms include immediate and retarded apoptosis (due to the generation of reactive oxygen species), neovascularization, activation of the FAS/FAS ligand system (which activates programmed cell death) and reduction in TGFβ, TNFα, IL-5, -6, -8, -13 and -31 and IFNγ [2]. Therefore, according to what we observed in our samples, it seems that on one hand, there is a reduction in the excess cellularity due to apoptosis, and on the other hand, there is an activation of the remaining fibroblasts which produces a rupture of the disorganized collagen due to the action of metalloproteinases and a new, denser and better organized collagen. Modulation of endothelial dysfunction induced by UVA-1, with an increase in CD34 expression [4], may also play a role, as endothelial dysfunction and abnormal blood vessel regulation have been proposed as pathogenic factors in keloid development [5]. In our samples, we observed an increase in vascular structures after the treatment. Finally, mucin, which is found at increased levels in keloids [6], was reduced after the UVA-1 sessions. All these mechanisms are responsible for the striking changes observed in the histological images of our case, which correlated with patient-reported outcomes. The aesthetic impairment barely changed, maybe because these changes occur mainly in the dermis instead of the epidermis; however, when the patient was asked, she said she was satisfied with the treatment.

There are various UVA-1 dosage regimen recommendations. Generally, each cycle consists of 15–20 sessions with increasing doses, and no more than two cycles per year are recommended [2]. In our case, the use of only one cycle of 25 sessions with a low-dosage regimen showed striking histological changes and an improvement in the patient’s symptoms. There have been reports of several skin diseases being successfully treated with UVA-1 phototherapy—for example, sclerotic diseases, such as systemic sclerosis and morphea, inflammatory conditions, such as eczema and prurigo, granulomatous diseases, such as sarcoidosis and annular granuloma, and T cell lymphomas and related disorders, such as pityriasis lichenoides and lymphomatoid papulosis [7]. To the best of our knowledge, there have only been two cases of keloids treated with UVA-1 laser [8] and a few cases treated with UVA-1 phototherapy, all with high doses [9,10,11], but no cases treated with a low-dosage regimen have been reported to date. UVA-1 phototherapy has less risk of burns and skin cancer development than other types of phototherapy [9], and the use of low doses further decreases this risk.

## 4. Conclusions

UVA-1 phototherapy can improve keloids both histologically and clinically and could be a first-line treatment for this challenging condition. Future research will determine the role of UVA-1 phototherapy in the management of keloids and the most appropriate dosages.
